# Biodegradation of Cyanide-Based Compounds by Rhodanese Produced from *Kocuria rhizophila* Under Submerged Fermentation and Its Role in Environmental Detoxification

**DOI:** 10.3390/molecules31060915

**Published:** 2026-03-10

**Authors:** Nada Z. Mahdi, Suhair Sh. Al-Siraj, Nehad A. Taher, Muneefah Abdullah Alenezi, Khyreyah J. Alfifi, Fauzeya Mateq Albalwe, Hanan Khalaf Anazi, Siham M. AL-Balawi, Mahmoud Galal, Maha F. Lotfy, Eman M. Sharaf

**Affiliations:** 1 Department of Biology, College of Science, Mustansiriyah University, Baghdad 10052, Iraq; 2 Department of Biology, Faculty of Science, University of Tabuk, Tabuk 71491, Saudi Arabia; 3 Department of Pharmaceutics, Faculty of Pharmacy, MSA University, Cairo 11799, Egypt; 4 Curative Care Sector, Ministry of Health and Population, Cairo 11841, Egypt; 5 Department of Agricultural Microbiology, Faculty of Agriculture, Ain Shams University, Cairo 11241, Egypt; 6 Bacteriology, Immunology and Mycology Department, Animal Health Research Institute, ARC, Shebin El Kom 32511, Egypt

**Keywords:** *Kocuria rhizophila*, rhodanese, submerged fermentation, cyanide detoxification

## Abstract

Widespread release of cyanide from industrial activities represents a significant environmental challenge due to its acute toxicity and adverse effects on biological systems. In response to this concern, this study focused on the production of rhodanese from *Kocuria rhizophila* under submerged fermentation conditions and the assessment of its relevance for cyanide detoxification applications. A soil-derived Gram-positive bacterium was isolated and identified as *Kocuria rhizophila* based on morphological traits, biochemical profile-based VITEK 2 analysis, and 16S rRNA gene sequencing. Preliminary screening confirmed rhodanese production with an activity of 0.968 RU/mL. Under cyanide-induced submerged fermentation, enzyme production followed a growth-associated pattern and reached maximal activity at 40 h under optimized conditions (35 °C, pH 8.0). Partial purification using sequential precipitation and chromatographic steps enhanced enzyme purity, and SDS–PAGE analysis of the final fraction revealed protein bands at approximately 40, 140, and 260 kDa. Biochemical characterization showed Km values of 33.9 mM for KCN and 19.7 mM for sodium thiosulfate, with a Vmax of ~5.6 µmol min^−1^ mL^−1^ for KCN and optimal activity at pH 7–8 and 35 °C. Functional assays demonstrated efficient cyanide detoxification, achieving >85% conversion of KCN, ~92% of NaCN, and 65–77% of Ca (CN)_2_ within 60 min in vitro. Collectively, these findings demonstrate that *Kocuria rhizophila* represents a promising microbial source of rhodanese with efficient cyanide-detoxifying activity, highlighting its potential for biotechnological and environmental remediation applications.

## 1. Introduction

Cyanide-based compounds represent a persistent environmental threat due to their high solubility, mobility in soil and groundwater, and acute toxicity toward aerobic organisms. Their widespread release is associated with agricultural activities, pesticide degradation, mining operations, and industrial processes, leading to contamination of terrestrial and aquatic ecosystems [1,2]. At the cellular level, cyanide exerts toxicity primarily through inhibition of cytochrome c oxidase, disrupting mitochondrial respiration and energy metabolism, which contributes to ecological imbalance and long-term risks to human health, particularly in contaminated agricultural and industrial regions [3,4]. Conventional remediation strategies, including oxidation and adsorption, are often constrained by high operational costs, incomplete detoxification, and the generation of secondary pollutants, driving increased interest in biological alternatives [5]. Enzyme-based bioremediation has therefore emerged as a sustainable approach capable of selectively transforming toxic compounds under mild conditions with minimal environmental burden [6]. Among cyanide-detoxifying enzymes, rhodanese catalyzes the conversion of cyanide into the less toxic thiocyanate via sulfane sulfur transfer reactions, representing an efficient biochemical route for cyanide detoxification [7,8]. Recombinant and native microbial rhodaneses have demonstrated effective cyanide removal in aqueous systems, often exhibiting higher efficiency than physicochemical treatments [9], and several exhibit tolerance to broad pH and temperature ranges, enhancing their applicability under environmentally relevant and industrial conditions [10,11]. Despite these advantages, research has largely focused on a limited group of well-established cyanide-degrading bacteria, including *Pseudomonas*, *Bacillus*, and *Klebsiella*. Although effective, these organisms may exhibit constraints related to stress sensitivity or reduced stability during prolonged cyanide exposure [12], highlighting the need to explore alternative microbial hosts with enhanced physiological robustness. *Kocuria rhizophila*, a Gram-positive actinobacterium commonly associated with soil and rhizosphere environments, has been reported to display notable resistance to oxidative stress, desiccation, osmotic pressure, and toxic compounds, supported by its adaptive metabolic traits [13,14]. Genome-based analyses further reveal that *K. rhizophila* harbors an extensive repertoire of catabolic and redox-related enzymes, indicating a strong intrinsic capacity for xenobiotic tolerance and transformation [15]. More broadly, Actinobacteria are widely recognized for their environmental resilience and metabolic versatility, yet remain underutilized as platforms for detoxification-related bioprocesses [16,17]. Submerged fermentation (SmF) provides an effective framework for exploring such alternative microbial enzyme sources, offering precise control over cultivation parameters, high reproducibility, and compatibility with large-scale enzyme production and downstream processing [18]. The present study therefore aimed to evaluate *Kocuria rhizophila* as a robust microbial source of rhodanese by investigating enzyme production under cyanide-induced submerged fermentation conditions, followed by partial purification, biochemical characterization, and assessment of its efficiency in detoxifying different cyanide-based compounds.

## 2. Results

### 2.1. Isolation of Kocuria rhizophila Isolates

A total of eleven bacterial isolates were recovered from soil samples following serial dilution and culturing on Brain Heart Infusion agar. Colonies showing distinct pigmentation and consistent morphology were selected during initial screening and purified by subculturing. One representative isolate was subsequently selected for detailed morphological, biochemical, and molecular identification.

### 2.2. Identification of Kocuria rhizophila Isolates

#### 2.2.1. Morphological-Based Identification

Microscopical examination of the selected isolate revealed coccoid cells that were predominantly spherical and arranged in pairs, tetrads, and irregular clusters. No elongated or filamentous forms were observed. Macroscopic evaluation showed circular colonies with smooth margins, convex elevation, and uniform pigmentation across replicate plates, allowing reliable preliminary differentiation of the isolate (Figure S1).

#### 2.2.2. VIETK-Based Biochemical Identification

Biochemical identification using the VITEK automated system generated a reproducible biochemical reaction profile for the isolate (Figure S2). Comparison of this profile with reference patterns in the system database resulted in identification of the isolate as *Kocuria rhizophila*, supporting the phenotypic observations obtained from morphological analyses.

#### 2.2.3. Molecular Identification and Phylogenetic Characterization of the Isolate

**Figure 1:**
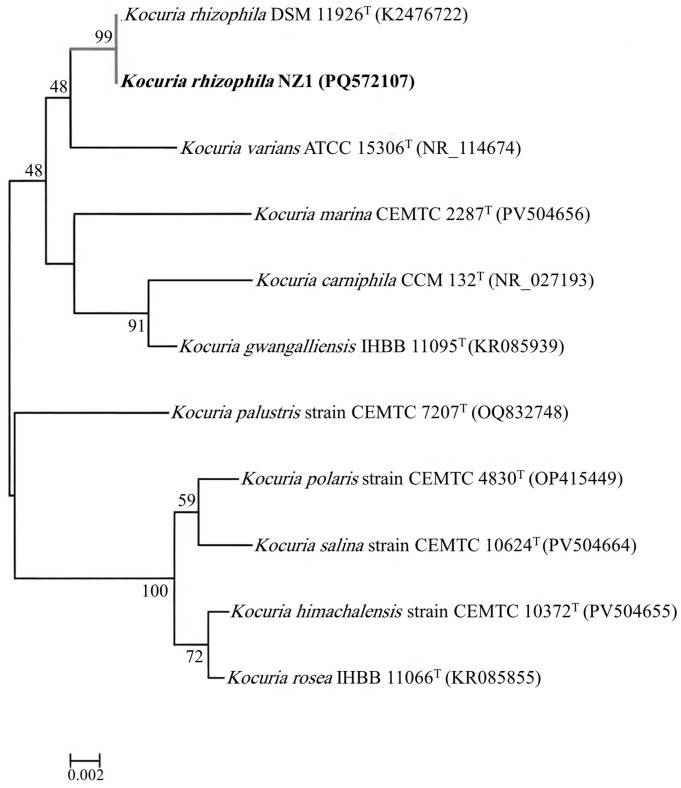
Phylogenetic tree based on 16S rRNA gene sequences showing the relationship between *Kocuria rhizophila* NZ1 and related *Kocuria* species retrieved from GenBank. Numbers at the nodes indicate bootstrap values (%), and the isolate obtained in this study is shown in bold. The scale bar represents 0.002 nucleotide substitutions per site.

PCR amplification of the 16S rRNA gene yielded a single amplicon of approximately 1485 bp, corresponding to the expected size of the bacterial 16S rRNA gene (Figure S3). Sequence alignment and pairwise comparison demonstrated high nucleotide similarity with reference *Kocuria rhizophila* sequences deposited in public databases (Figures S4 and S5). The obtained 16S rRNA gene sequence was deposited in the GenBank database under accession number PQ572107. Phylogenetic analysis based on 16S rRNA gene sequences showed clustering of the isolate within the *K. rhizophila* clade with strong bootstrap support, confirming its species-level taxonomic assignment (Figure 1).

### 2.3. Rhodanese-Producing Capacity of Kocuria rhizophila

Among the cyanide-tolerant colonies identified during the initial screening process, only one isolate exhibited measurable rhodanese activity under the applied assay conditions. This isolate, identified as *Kocuria rhizophila*, demonstrated sustained growth under cyanide-induced conditions, as reflected by a cell dry weight of 0.0642 g per 100 mL culture, confirming its ability to tolerate and proliferate in the presence of cyanide. The corresponding rhodanese activity of 0.968 RU mL^−1^ verified active enzyme formation and distinguished this isolate from other screened colonies that did not exhibit measurable activity. Based on its confirmed rhodanese-producing capability, this *K. rhizophila* isolate was selected for subsequent production profiling, optimization of fermentation conditions, and biochemical characterization.

### 2.4. Production Profile of Rhodanese Enzyme by Kocuria rhizophila

**Figure 2:**
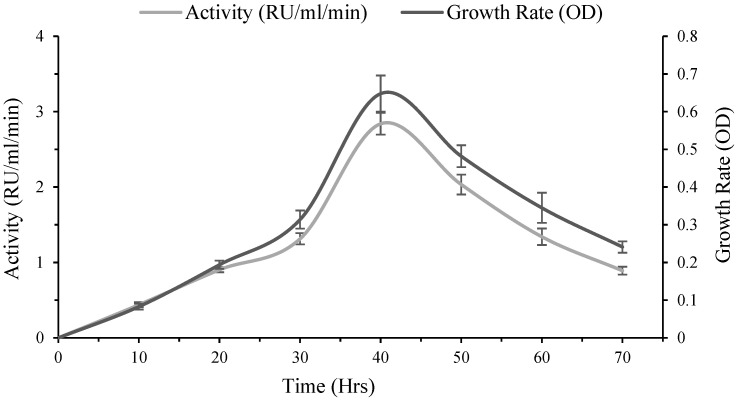
*Kocuria rhizophila* growth rate and concomitant rhodanese activity observed during incubation period.

Time-course monitoring revealed a clear production pattern in which rhodanese activity and biomass increased to a distinct maximum before declining at later stages. All experiments were performed in triplicate, and values represent mean ± SD. Enzyme activity rose progressively from 0.00 RU/mL/min at 0 h to a peak of 2.84 RU/mL/min at 40 h, followed by a reduction to 2.034 RU/mL/min at 50–60 h and a sharper decrease to 0.8915 RU/mL/min at 70 h. Cell dry weight similarly increased to its highest value at 40 h (0.0462 g/100 mL) and then decreased gradually thereafter. The growth rate (OD) showed a marked increase up to 40 h (0.648) followed by a decline, indicating that maximum enzyme yield coincided with the highest recorded biomass and OD at 40 h (Figure 2).

### 2.5. Influence of Variable Fermentation Conditions on Enzyme Production

All experiments were performed in triplicate, and data are presented as mean ± SD.

#### 2.5.1. Influence of Substrate Concentration on Enzyme Yield

**Figure 3:**
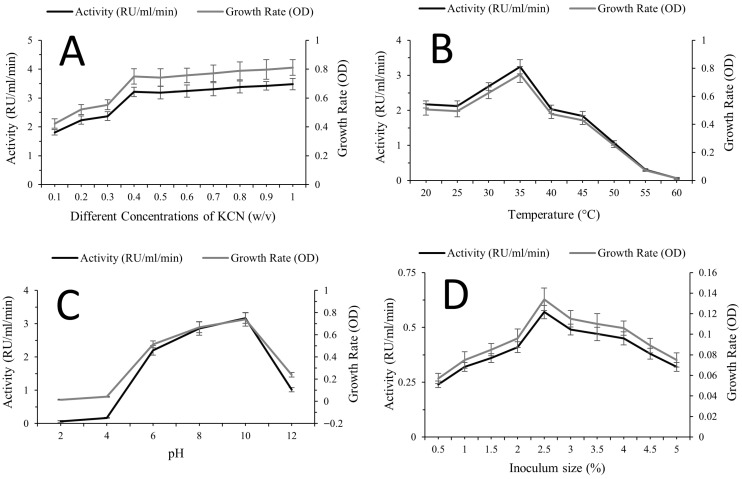
Effects of fermentation conditions on rhodanese production and growth of *Kocuria rhizophila*. (**A**) Influence of KCN concentration on enzyme yield and growth rate. (**B**) Influence of temperature on enzyme yield and growth rate. (**C**) Influence of initial pH on enzyme yield and growth rate. (**D**) Influence of inoculum size on enzyme yield and growth rate.

Rhodanese production increased progressively with rising KCN concentration, showing a clear positive relationship between substrate level, enzyme yield, and growth rate (Figure 3A). Enzyme activity increased from 1.81 RU/mL/min at 0.1% to 3.21 RU/mL/min at 0.4%, with continued incremental increases reaching the highest recorded value at 1.0% (3.48 RU/mL/min). A minor fluctuation was observed at 0.5% (3.18 RU/mL/min) relative to 0.4%, but the overall trend remained upward. Notably, growth rate (OD) followed a similar increasing trend across the tested concentrations, indicating that enhanced rhodanese production under elevated KCN levels was associated with improved cellular proliferation within the investigated range (Figure 3A).

#### 2.5.2. Influence of Temperature on Enzyme Yield

Temperature markedly influenced rhodanese production, with enzyme activity increasing to an optimum before declining sharply at higher temperatures (Figure 3B). Activity rose from 2.17 RU/mL/min at 20 °C to 2.68 RU/mL/min at 30 °C, reaching the maximum value at 35 °C (3.24 RU/mL/min). Beyond this point, activity decreased to 2.03 RU/mL/min at 40 °C and declined further to 0.31 RU/mL/min at 55 °C and 0.06 RU/mL/min at 60 °C. The growth rate (OD) exhibited a parallel trend, peaking at 35 °C and decreasing progressively at higher temperatures. This concurrent decline in both enzyme yield and growth above the optimum indicates that rhodanese production is closely associated with cellular growth and is negatively affected by thermal stress beyond the optimal temperature (Figure 3B).

#### 2.5.3. Influence of pH on Enzyme Yield

Initial medium pH significantly influenced rhodanese production, with enzyme activity increasing from acidic to alkaline conditions before declining at the highest tested pH (Figure 3C). Activity was minimal at pH 2 (0.06 RU/mL/min) and increased markedly at pH 6 (2.20 RU/mL/min) and pH 8 (2.86 RU/mL/min), reaching a maximum at pH 10 (3.17 RU/mL/min). A decline was observed at pH 12 (1.02 RU/mL/min). The growth rate (OD) followed a similar trend, rising progressively toward pH 10 and decreasing at pH 12. This parallel pattern indicates that optimal rhodanese production occurred under alkaline conditions that also supported maximal cellular growth, suggesting growth-associated enzyme synthesis within the tested pH range (Figure 3C).

#### 2.5.4. Influence of Initial Inoculum Concentration on Enzyme Yield

Initial inoculum size exhibited a defined optimum effect on rhodanese production (Figure 3D). Enzyme activity increased from 0.24 RU/mL/min at 0.5% to 0.41 RU/mL/min at 2.0%, reaching a maximum at 2.5% (0.57 RU/mL/min). Beyond 2.5%, activity declined progressively, decreasing to 0.49 RU/mL/min at 3.0% and further to 0.32 RU/mL/min at 5.0%. The growth rate (OD) followed a comparable pattern, increasing with inoculum size up to 2.5% and declining at higher percentages. This parallel response indicates that optimal enzyme production coincided with maximal cellular proliferation, while excessive inoculum levels likely imposed physiological constraints that limited both growth and rhodanese yield (Figure 3D).

### 2.6. Enzyme Purification

The purification profile demonstrated progressive enrichment of rhodanese activity across successive fractionation steps. The crude extract exhibited a specific activity of 2.17 µmol/mg/min (1.00-fold). Acetone precipitation increased the specific activity to 3.92 µmol/mg/min, corresponding to approximately 1.81-fold purification. Ammonium sulphate fractionation resulted in a specific activity of 2.82 µmol/mg/min, reflecting partial redistribution of proteins during salt precipitation. Subsequent ion-exchange chromatography (CM-Sephadex C-50) further enriched the enzyme, increasing the specific activity to 9.83 µmol/mg/min. The final gel filtration step (Sephadex G-100) produced the highest specific activity (11.56 µmol/mg/min), corresponding to the greatest purification fold reported in this study. Overall, these results confirm stepwise enhancement of rhodanese purity, with maximal enrichment achieved following gel filtration (Table 1).

### 2.7. Rhodanese Enzyme Extract Characterization

#### 2.7.1. Determination of Kinetic Parameters

Kinetic analysis revealed substrate-dependent differences in the apparent affinity of rhodanese toward potassium cyanide (KCN) and sodium thiosulfate (Na_2_S_2_O_3_). The apparent Km value for KCN (33.92 ± 0.11 mM) was higher than that obtained for Na_2_S_2_O_3_ (19.65 ± 0.24 mM), indicating a lower affinity for KCN, while Vmax values were comparable for both substrates (Table 2). Lineweaver–Burk double-reciprocal plots showed linear relationships for both substrates, supporting the reliability of the kinetic parameters. Varying KCN concentrations (0.005–0.05 M) at a fixed Na_2_S_2_O_3_ concentration produced a linear plot of 1/v versus 1/[S], whereas varying Na_2_S_2_O_3_ concentrations under fixed KCN conditions yielded a comparable linear relationship. The steeper slope observed in the KCN plot is consistent with its higher Km value.

#### 2.7.2. Effect of pH on Enzyme Activity

**Figure 4:**
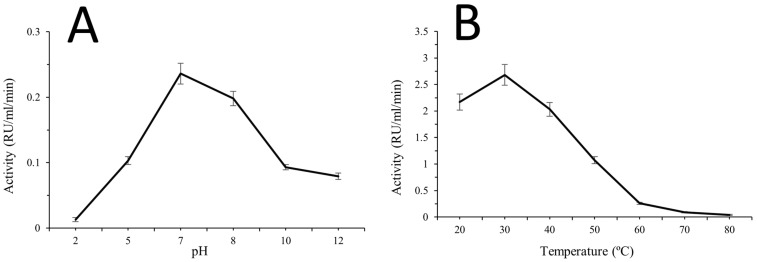
Effects of physicochemical conditions on rhodanese activity. (**A**) Effect of pH. (**B**) Effect of temperature.

Enzyme activity varied across the tested pH range, with a clear maximum observed at neutral pH followed by reduced activity under more alkaline conditions. Activity increased from 0.013 RU/mL/min at pH 2 to 0.103 at pH 5 and reached the highest value at pH 7 (0.236 RU/mL/min). Activity then decreased to 0.198 at pH 8 and declined further at pH 10 (0.093) and pH 12 (0.079). This profile indicates that rhodanese activity increased with pH up to pH 7 and then showed an inverse relationship with pH beyond the optimum (Figure 4A).

#### 2.7.3. Effect of Temperature on Enzyme Activity

Temperature-dependent activity assessment showed higher enzyme activity at lower temperatures, followed by progressive reduction as temperature increased. Activity was highest at 20 °C (2.17 units mL^−1^) and decreased at 40 °C (2.03), with a further marked decline at 50 °C (1.07). At elevated temperatures, activity continued to drop, reaching 0.26 at 60 °C and 0.04 at 80 °C. The observed pattern indicates an inverse relationship between temperature and rhodanese activity across the higher temperature range tested (Figure 4B).

#### 2.7.4. Effect of Metal Ions

Metal ions modulated rhodanese activity in a concentration-dependent manner (Table 3). At 1 mM, KCl caused the strongest inhibition (70.75 ± 0.35%), while MnCl_2_ (78.93 ± 0.39%) and BaCl_2_ (80.20 ± 0.28%) showed moderate reductions. MgCl_2_ (92.15 ± 0.21%), NaCl (88.46 ± 0.20%), and SnCl_2_ (87.75 ± 0.35%) maintained activity closer to the control. Increasing the concentration to 5 mM revealed clearer divergence: NaCl declined sharply to 45.76 ± 0.24%, indicating strong dose-dependent inhibition, whereas KCl (64.19 ± 0.87%) and MgCl_2_ (82.16 ± 0.20%) showed gradual decreases. In contrast, NiCl_2_ and MnCl_2_ exhibited activation trends, rising to 91.0 ± 0.38% and 88.64 ± 0.76%, respectively, while BaCl_2_ shifted toward enhanced activity (87.32 ± 0.94%), suggesting a biphasic effect. At 10 mM, NaCl showed severe inhibition (18.50 ± 0.31%), whereas KCl and MgCl_2_ declined moderately (61.05 ± 0.32% and 75.00 ± 0.65%). Conversely, SnCl_2_ (98.58 ± 0.10%), NiCl_2_ (95.0 ± 1.02%), and MnCl_2_ (93.81 ± 0.98%) demonstrated progressive activation. Overall, rhodanese activity exhibited distinct ion-specific patterns, including strong dose-dependent inhibition (NaCl), moderate inhibition (KCl, MgCl_2_), and concentration-dependent activation or biphasic responses (SnCl_2_, NiCl_2_, MnCl_2_, BaCl_2_).

#### 2.7.5. Substrate Specificity and Kinetics

Substrate specificity testing demonstrated that sodium thiosulfate supported the highest relative activity, while alternative sulfur-containing compounds resulted in substantially lower specificity percentages. Sodium thiosulfate (Na_2_S_2_O_3_) was set as 100.0 ± 0.00%, whereas sodium metabisulfite (Na_2_S_2_O_5_) yielded 15.4 ± 0.28% and ammonium persulfate ((NH_4_)_2_S_2_O_8_) showed 24.15 ± 0.21%. 2-mercaptoethanol exhibited 18.0 ± 0.00%, and sodium sulfite resulted in 19.80 ± 0.14%. These results indicate a strong preference toward sodium thiosulfate relative to the other evaluated sulfur compounds under the same assay conditions (Table 4).

#### 2.7.6. SDS-PAGE Protein Profiling

**Figure 5:**
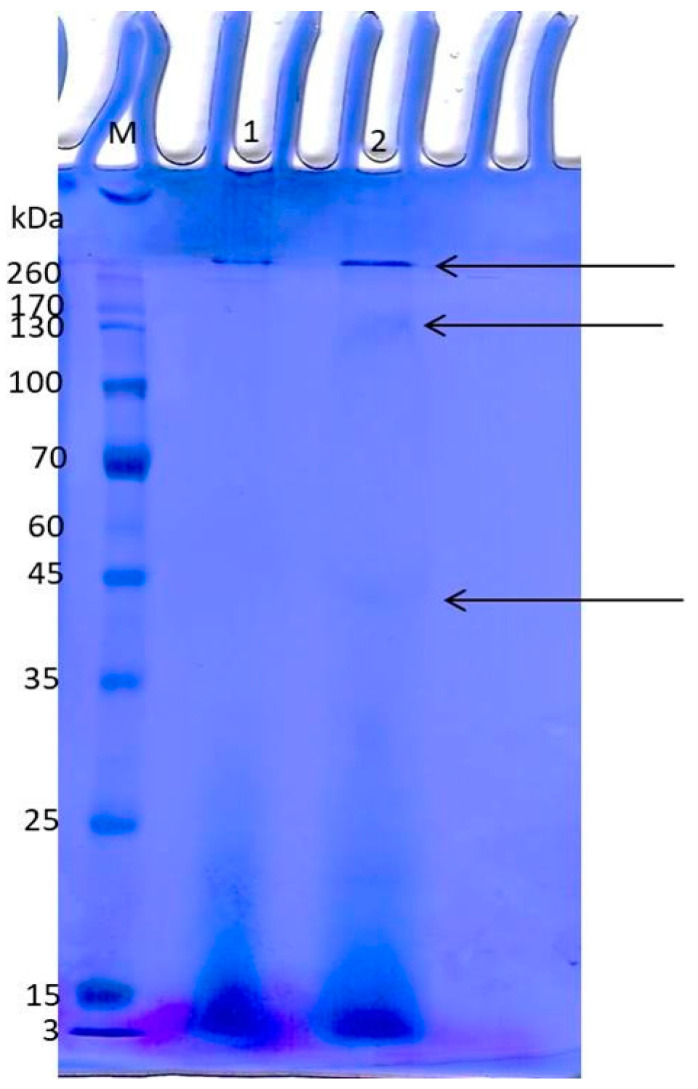
SDS–PAGE profile of partially purified rhodanese from *Kocuria rhizophila*. Lane M: molecular weight marker. Lane 2: purified fraction showing protein bands. Arrows indicate protein bands observed at approximately 40 kDa, 140 kDa, and 260 kDa.

SDS–PAGE analysis of the partially purified rhodanese preparation revealed multiple protein bands with apparent molecular masses of approximately 40 kDa, 140 kDa, and 260 kDa. The band observed near 40 kDa corresponds to the expected molecular mass range reported for bacterial rhodaneses. The higher-molecular-weight bands may represent oligomeric or aggregated forms of the enzyme that persisted despite denaturing conditions. The presence of more than one band indicates that the preparation represents a partially purified enzyme fraction rather than a homogenous protein sample. Nevertheless, enzymatic activity measurements confirmed that rhodanese activity was retained in the purified fraction (Figure 5).

### 2.8. Biodegradation Potential of Cyanide-Based Pesticides by Rhodanese Enzyme

**Figure 6:**
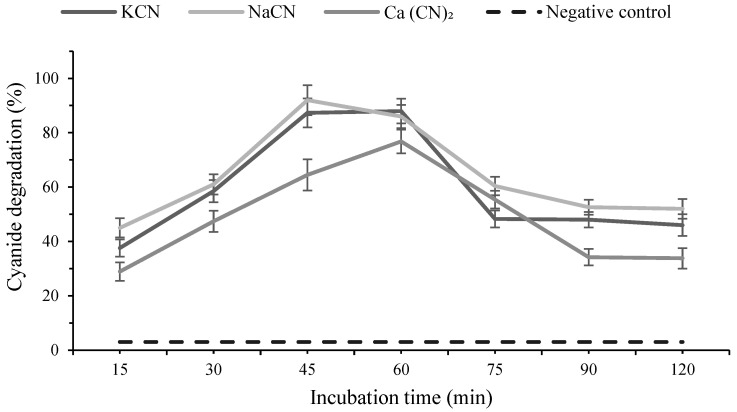
Time-course of rhodanese-mediated biodegradation of cyanide-based compounds in the presence of an active enzyme and a negative control.

Rhodanese treatment showed time-dependent changes in cyanide degradation percentages across the tested cyanide sources, with degradation increasing to a maximum and then decreasing at later time points. For KCN, degradation increased from 37.6% at 15 min to 87.3% at 45 min and reached 88.0% at 60 min, followed by a decline to approximately 48–46% from 75 to 120 min. A similar pattern was observed for NaCN, which rose from 45.0% at 15 min to a maximum of 92.0% at 45 min, then decreased to approximately 52% by 90–120 min. For Ca(CN)_2_, degradation increased from 28.9% at 15 min to 76.8% at 60 min and then declined thereafter. In contrast, the negative control (no enzyme) showed no appreciable change in cyanide levels throughout the incubation period, confirming that the observed degradation was enzyme-dependent. Across all three cyanide forms, the data indicate an initial direct relationship between incubation time and degradation up to peak values (45–60 min), followed by a reduction in degradation efficiency at extended incubation times beyond the peak (Figure 6).

## 3. Discussion

Cyanide contamination from mining and industrial activities poses severe ecological and toxicological risks owing to its high solubility and acute toxicity [19], prompting increasing interest in enzyme-based detoxification as a sustainable remediation strategy capable of converting cyanide into less toxic compounds under environmentally compatible conditions [20]. In this study, rhodanese production by the soil-derived Gram-positive bacterium *Kocuria rhizophila* was investigated under submerged fermentation to assess its potential role in cyanide biodegradation. Morphological and biochemical characteristics, including Gram-positive non-motile cocci arranged in tetrads and sulfur-yellow colonies on Brain Heart Infusion agar, were consistent with the original description of *K. rhizophila* [21,22], while accurate identification was ensured using the VITEK 2 Gram-positive system combined with 16S rRNA gene sequencing, a validated approach widely applied in previous studies [23,24,25]. Preliminary screening confirmed rhodanese production by *K. rhizophila*, as evidenced by measurable enzyme activity (0.968 RU mL^−1^) and biomass formation. The limited number of reports describing rhodanese production within Kocuria highlights the novelty of this finding compared with established producers such as Klebsiella and Bacillus [11,26,27], although rhodanese production has been reported in environmental and industrial effluent isolates, including bacteria from bio-oxidation systems [28]. Under submerged fermentation, rhodanese production followed a growth-associated pattern, peaking at 40 h before declining, consistent with reports for Pseudomonas aeruginosa, Bacillus brevis, and Klebsiella oxytoca, where maximal enzyme synthesis typically coincides with the late exponential or early stationary phase [29,30]. Enzyme yield was strongly influenced by substrate concentration, temperature, pH, and inoculum size, with optimal production occurring at moderate mesophilic temperature (35 °C) and alkaline pH, followed by declines at higher values due to substrate inhibition, thermal stress, nutrient depletion, and oxygen limitation, as reported for other microbial rhodaneses produced via submerged cultivation [31,32]. Importantly, the divergence between optimal conditions for rhodanese production and those for enzymatic activity reflects a common distinction between microbial enzyme biosynthesis and catalytic performance, as production is governed by cellular physiology and stress-induced regulatory mechanisms, whereas activity optima are determined by intrinsic protein stability and catalytic chemistry, a phenomenon widely reported for rhodaneses and other microbial enzymes [11,29,31,33]. The multistep purification strategy employed, including acetone precipitation, ammonium sulfate fractionation, and chromatographic separation, follows a classical and reliable approach for microbial enzyme purification [34,35]. Kinetic characterization revealed Km values in the 10^−2^ M range, with apparent affinities of 33.9 mM for KCN and 19.7 mM for sodium thiosulfate and a Vmax of approximately 5.6 µmol min^−1^ mL^−1^, comparable to values reported for B. licheniformis and K. oxytoca, indicating broadly conserved sulfurtransferase behavior across bacterial genera [33,36]. Although the relatively high Km for KCN indicates moderate substrate affinity, this does not preclude practical applicability, as rhodanese-mediated detoxification can proceed efficiently at sub-saturating cyanide concentrations when sufficient enzyme levels are present, and millimolar cyanide concentrations are commonly encountered in industrial effluents and mining leachates [19,27,33,36]. The enzyme exhibited optimal activity at pH 7–8, retained functionality under alkaline conditions, and remained stable at elevated temperatures and in the presence of common metal ions, supporting its robustness for environmental and biotechnological applications [11,33]. SDS–PAGE analysis of the partially purified fraction revealed protein bands at approximately 40 kDa together with higher-molecular-weight species (~140 and ~260 kDa). The band near 40 kDa falls within the molecular mass range commonly reported for bacterial rhodaneses (typically 30–40 kDa) [33,36], while the higher bands may reflect oligomeric assemblies or incomplete dissociation under denaturing conditions. Functionally, *K. rhizophila* rhodanese achieved high cyanide detoxification efficiencies (>85% for KCN and 92% for NaCN within 60 min) and effectively degraded insoluble calcium cyanide, in agreement with established sulfurtransferase-mediated detoxification pathways [10,37]. The decline in degradation efficiency observed after 45–60 min likely reflects time-dependent biochemical constraints, including partial enzyme inactivation under prolonged cyanide exposure [27,38], product inhibition by accumulated thiocyanate or persulfide intermediates [9,27], and evolving reaction conditions such as limited sulfur-donor availability or local pH shifts [31,39], while the formation of secondary cyanide complexes may further reduce measurable free cyanide at extended incubation times [1,19]. Collectively, these factors provide a mechanistic explanation for the observed time-course behavior and highlight the dynamic nature of rhodanese-mediated cyanide detoxification systems.

## 4. Materials and Methods

### 4.1. Isolation of Kocuria rhizophila from Soil

#### 4.1.1. Soil Sampling

Surface soil samples (10 cm depth) were aseptically collected from an agricultural site with a known history of pesticide application. Samples were transferred into sterile polyethylene bags, then labeled and transported to the laboratory for immediate analysis. Moisture content was minimized by keeping samples on ice during transport. Soil sampling followed standard microbiological guidelines for environmental bacterial isolation [40].

#### 4.1.2. Serial Dilution and Culturing on Brain Heart Infusion Agar

Ten grams of soil from each sample were gently mixed with 90 mL of sterile physiological saline, then diluted stepwise to a final dilution of 10^−6^. From each dilution, 100 µL was plated onto Brain Heart Infusion agar and left to incubate between 30 and 37 °C for roughly 24 to 48 h. Colonies with noticeable pigmentation and colony shapes that, at least visually, aligned with known Kocuria characteristics were picked and subcultured for purification. Preparation of media and all cultivation steps followed commonly accepted protocols used in routine environmental microbiology work [41].

#### 4.1.3. Inoculum Preparation

Presumptive *Kocuria* isolates were transferred to fresh BHI broth and incubated at 30 °C with agitation at 150 rpm to obtain log-phase cells. The resulting cultures were adjusted to a 0.5 McFarland standard using a densitometer (DEN-1, Biosan, Rīga, Latvia) to standardize inoculum density before further characterization. Inoculum standardization followed accepted microbial inoculum preparation protocols [42].

### 4.2. Identification of Kocuria rhizophila Isolates

#### 4.2.1. Macroscopic and Microscopic Examination

Isolates were initially differentiated on the basis of colony characteristics, including pigmentation, form, margin, elevation, and surface texture. Gram staining was performed using standard reagents (Oxoid, Basingstoke, UK), and cellular morphology was examined under a light microscope (Olympus CX23, Tokyo, Japan). *Kocuria* isolates were expected to present as Gram-positive cocci occurring in tetrads or clusters. Morphological assessment followed CLSI-recommended microbiological characterization procedures [42].

#### 4.2.2. VITEK-Based Biochemical Identification

Biochemical profiling was conducted using the VITEK^®^ 2 Compact system (bioMérieux, Craponne, France) with GP identification cards according to the manufacturer’s specifications. Pure cultures were suspended in 0.45% NaCl to a 0.5 McFarland turbidity and loaded into the instrument. Automated biochemical identification outputs were compared to the VITEK reference database for species-level resolution. This automated system is widely validated for high-throughput identification of Gram-positive cocci [43].

#### 4.2.3. Molecular Identification via 16S rRNA Gene Amplification and Sequencing

Genomic DNA was isolated from the bacterial cultures using the QIAamp DNA Mini Kit (Qiagen GmbH, Hilden, Germany), following the supplier’s instructions without modification. Amplification of the 16S rRNA gene relied on the universal primers 27F and 1492R, set up in a 25 µL reaction mixture that included EmeraldAmp Max PCR Master Mix (Takara, Gunma, Japan), 1 µL of each primer at 20 pmol, 5.5 µL of nuclease-free water, and 5 µL of the extracted DNA. PCR amplification was carried out on an Applied Biosystems 2720 thermal cycler (Applied Biosystems, Foster City, CA, USA), beginning with denaturation at 95 °C for 5 min, followed by 35 cycles of 95 °C for 30 s, annealing at 55 °C for 30 s, and extension at 72 °C for 90 s, then ending with a final extension step at 72 °C for 7 min. The resulting amplicons were resolved on a 1.5% agarose gel (Applichem GmbH, Darmstadt, Germany) prepared in 1× TBE buffer and examined using a gel documentation system (Alpha Innotech, Biometra, Göttingen, Germany). PCR products were subsequently purified with a commercial cleanup kit and sequenced using the Sanger approach. Chromatograms were inspected for quality, after which the sequences were compared against the NCBI GenBank database using BLASTn (v2.14.1+). Phylogenetic relationships were inferred in MEGA X through the neighbor-joining method, supported by 1000 bootstrap replications [44,45].

### 4.3. Evaluation of the Rhodanese-Producing Potential of Kocuria rhizophila

Environmental bacterial isolates were initially screened for cyanide tolerance on modified Bushnell–Haas medium supplemented with 0.3% (*w*/*v*) potassium cyanide (KCN). Colonies exhibiting sustained growth under these selective conditions were considered cyanide-tolerant and subjected to further screening for rhodanese production. Each tolerant isolate was inoculated into 100 mL of basal broth containing 0.3% (*w*/*v*) KCN and 0.5% (*w*/*v*) yeast extract, adjusted to pH 9.5, and incubated at 30 °C with shaking (≈150 rpm) for 48 h. Following incubation, cells were harvested by centrifugation (10,000× *g*, 10 min, 4 °C), washed with phosphate buffer (pH 7.5), and disrupted by sonication on ice to obtain crude cell-free extracts. Rhodanese (thiosulfate–cyanide sulfurtransferase) activity was quantified using the thiocyanate-formation colorimetric assay based on ferric–thiocyanate complex formation, following a validated protocol for bacterial sulfurtransferases involved in cyanide detoxification [27]. The isolate exhibiting measurable rhodanese activity under these screening conditions was selected for subsequent production, optimization, and characterization studies.

### 4.4. Rhodanese Production Under Submerged Fermentation

Submerged fermentation was conducted in 250 mL Erlenmeyer flasks containing 100 mL of modified Luria–Bertani medium composed of peptone (3% *w*/*v*), NaCl (0.5% *w*/*v*), and yeast extract (0.5% *w*/*v*), with the initial medium pH adjusted to 9.5 prior to sterilization. The basal medium was autoclaved at 121 °C for 15 min. A sterile potassium cyanide (KCN) solution, prepared separately and membrane-filtered (0.22 μm), was aseptically added after cooling to achieve a final concentration of 0.3 mM, serving as an inducer for rhodanese production. All cyanide-related procedures were carried out in a certified chemical fume hood using appropriate personal protective equipment, and cyanide-containing wastes were chemically detoxified in accordance with institutional biosafety and chemical safety regulations prior to disposal. The flasks were inoculated with 1 mL of a standardized *Kocuria rhizophila* suspension adjusted to an optical density of OD_600_ = 0.8 (1 × 10^8^ CFU/mL) and incubated at 37 °C with agitation at 170 rpm. Fermentation was continued for approximately 70 h (nearly four days), during which samples were withdrawn at 10 h intervals for the determination of rhodanese activity and cell dry weight, enabling monitoring of enzyme production throughout the cultivation period. At each sampling point, cultures were centrifuged at 11,000× *g* for 10 min at 4 °C, and the cell-free supernatants were collected for subsequent analysis [36].

### 4.5. Optimization of Rhodanese Production Under Variable Fermentation Conditions

#### 4.5.1. Influence of Substrate Concentration on Enzyme Yield

To explore how starting cyanide levels shape rhodanese production, the culture medium was supplemented with potassium cyanide at concentrations ranging from 0.1 to 1.0% (*w*/*v*), increasing in steps of 0.1%. For each condition, 250 mL Erlenmeyer flasks holding 100 mL of production medium were set to pH 9.5, seeded with 1% (*v*/*v*) of a standardized *K. rhizophila* inoculum, and placed at 37 °C with shaking at 170 rpm for 48 h. At specific time points, culture samples were collected and clarified by centrifugation at 10,000× *g* for 10 min at 4 °C. The supernatants were then analyzed for rhodanese activity alongside total protein content, allowing enzyme productivity to be evaluated across the different KCN concentrations [46].

#### 4.5.2. Influence of Temperature on Enzyme Yield

To assess the effect of temperature on enzyme biosynthesis, submerged fermentations were conducted at 20, 25, 30, 35, 40, 45, 50, 55 and 60 °C under otherwise identical cultivation conditions. Each flask received 1% (*v*/*v*) inoculum and was incubated at the designated temperature with agitation at 170 rpm for 48 h. Culture samples were clarified by centrifugation and analyzed for rhodanese activity and protein concentration. Optimal production temperature was identified based on maximal specific enzyme productivity [47].

#### 4.5.3. Influence of pH on Enzyme Yield

To assess how external pH shapes rhodanese synthesis, the production medium was set to pH 2, 4, 6, 8, 10, or 12 before sterilization, using suitable 50 mM buffer systems. Glycine–HCl was used for pH 2, acetate for pH 4, phosphate for pH 6, Tris for pH 8, carbonate–bicarbonate for pH 10, and glycine–NaOH for pH 12. Each flask received 1% (*v*/*v*) of a standardized culture and was incubated at 37 °C with agitation at 170 rpm for 48 h. After incubation, cultures were centrifuged to obtain cell-free supernatants, which were then examined for rhodanese activity and total protein content in order to estimate specific productivity across the tested pH range [48].

#### 4.5.4. Influence of Initial Inoculum Concentration on Enzyme Yield

The effect of inoculum size on rhodanese production was evaluated using the basal medium supplemented with 0.3 mM KCN. A pre-culture of *Kocuria rhizophila* grown in BHI broth to mid-log phase was used to inoculate production flasks at 0.5, 1.0, 1.5, 2.0, 2.5, 3.0, 3.5, 4.0, 4.5, and 5.0% (*v*/*v*). Each flask (100 mL working volume) was incubated at 37 °C and 170 rpm for 48 h. Samples were collected, centrifuged, and the clarified supernatants were analyzed for rhodanese activity and protein concentration to determine enzyme productivity at each inoculum level. This procedure follows standard inoculum-optimization approaches used in submerged fermentation studies [49].

### 4.6. Rhodanese Activity Assay and Protein Quantification

Rhodanese activity was determined using a modified thiosulfate: cyanide sulfurtransferase assay adapted from established protocols measuring thiocyanate formation and colorimetric detection [38]. Each reaction was assembled with 500 µL of borate buffer at pH 9.4, followed by 200 µL of 250 mM Na_2_S_2_O_3_, 200 µL of 250 mM KCN, and finally 100 µL of the enzyme extract. The mixture was left to react for 1 min at room temperature, then promptly stopped by adding 500 µL of 15% formaldehyde. Color formation was initiated by the addition of 1.5 mL of Sorbo reagent, after which absorbance was read at 460 nm on a Shimadzu UV-1800 spectrophotometer. Protein levels were estimated separately using the Bradford method, with bovine serum albumin serving as the calibration standard [50].

### 4.7. Crude Enzyme Extract Purification

#### 4.7.1. Acetone Precipitation

The crude supernatant was combined with nine volumes of ice-cold acetone and kept at −20 °C for about 60 to 90 min, allowing proteins to come out of solution. The resulting precipitate was recovered by centrifugation at 15,000 rpm for 15 min at 4 °C, then briefly air-dried before being redissolved in 0.1 M Tris–HCl buffer at pH 7.0. This acetone-based step followed routine protein concentration practices commonly used during preliminary enrichment [51].

#### 4.7.2. Ammonium Sulfate Fractionation

Ammonium sulfate precipitation was performed to concentrate and partially purify the enzyme extract from the crude supernatant. Solid ammonium sulfate was gradually added to achieve 85% saturation while maintaining the sample on ice with gentle stirring to prevent localized denaturation. The mixture was allowed to equilibrate overnight at 4 °C to ensure complete precipitation. Protein pellets were collected by centrifugation at 12,000 rpm for 30 min at 4 °C, briefly drained, and resuspended in 0.1 M phosphate buffer (pH 6.5). The resulting fraction was subsequently evaluated for rhodanese activity and total protein content [52].

#### 4.7.3. Ion-Exchange Chromatography (CM-Sephadex C-50)

After activation with successive washes of 0.1 M HCl and 0.1 M NaOH, the CM-Sephadex C-50 resin was equilibrated in phosphate buffer at pH 6.5. A 10 mL portion of the enzyme preparation was then loaded onto a column measuring 2.5 × 40 cm and rinsed with 0.5 M NaCl to clear away proteins that failed to bind. Elution of the retained proteins was achieved using 1.0 M NaCl at a flow rate of 20 mL/h, with fractions collected in 5 mL volumes for subsequent rhodanese activity analysis. The ion-exchange step followed well-established chromatographic practices used for enzyme purification [53].

#### 4.7.4. Gel Filtration Chromatography (Sephadex G-100)

Sephadex G-100 resin was hydrated in distilled water and equilibrated overnight at 4 °C; thereafter, the swollen gel was packed into a 2.5 × 90 cm column. The dialyzed enzyme solution (7 mL) was gently applied to the top of the column, and elution was carried out under gravity (or low-pressure flow) at a flow rate of approximately 10–15 mL/h to enhance resolution. Two-milliliter fractions were collected, and eluted proteins were monitored by absorbance at 280 nm or via rhodanese activity assay. Elution buffer consisted of 0.1 M phosphate (or Tris–HCl) buffer at pH 7.0–7.5 with minimal ionic strength to preserve enzyme stability. This gel-filtration step exploits size-exclusion principles, in which larger proteins elute first while smaller ones penetrate the bead pores and elute later, in accordance with established protocols for size-exclusion chromatography in protein purification [54].

### 4.8. Characterization of Partially Purified Rhodanese

#### 4.8.1. Determination of Kinetic Parameters

Kinetic constants (Km and Vmax) were determined by varying KCN concentrations (0.005–0.05 M) at a fixed Na_2_S_2_O_3_ concentration, and vice versa. Initial velocities were plotted using Lineweaver–Burk double-reciprocal plots. The Lineweaver–Burk method is a classical and widely accepted enzymological approach [55].

#### 4.8.2. Effect of pH on Enzyme Activity

Enzyme assays were conducted in buffers ranging from pH 3–11, including citrate (pH 3–5), phosphate (pH 6–8), and borate (pH 9–11). Each buffer replaced the standard assay buffer to evaluate pH-dependent activity changes [56].

#### 4.8.3. Effect of Temperature on Enzyme Activity

Reactions were carried out at temperatures from 30–80 °C at 10 °C increments. Substrates were pre-equilibrated at each temperature for 10 min before adding the enzyme. Temperature effects were evaluated following validated thermodynamic assessment procedures [33].

#### 4.8.4. Effect of Metal Ions

The modulatory effect of Mg^2+^, Ca^2+^, Hg^2+^, Na^+^, Ba^2+^, and K^+^ ions at concentrations of 1 mM, 5 mM, and 10 mM was assessed by incorporating metal salts into the standard reaction mixture, then the enzymatic inhibition or activation patterns were determined following the procedure [57].

#### 4.8.5. Substrate Specificity and Kinetics

Alternative sulfur donors, including sodium sulfite, 2-mercaptoethanol, ammonium persulfate, ammonium sulfate, and sodium metabisulfite, were substituted for thiosulfate in the assay. Relative activities were calculated using thiosulfate as the control [58].

#### 4.8.6. SDS-PAGE Protein Profiling

Protein patterns were examined by SDS–PAGE following the procedure described by Laemmli [59]. Each sample was combined with 5× loading buffer containing SDS, glycerol, and β-mercaptoethanol, briefly boiled for 5 min, and then separated on 12% resolving gels using a Bio-Rad Mini-PROTEAN apparatus. After electrophoresis, gels were stained with Coomassie Brilliant Blue G-250 and slowly destained until discrete banding became easy to distinguish. Apparent molecular weights were approximated by comparison with a pre-stained protein marker.

### 4.9. Evaluation of Rhodanese-Mediated Cyanide Pesticide Degradation

#### 4.9.1. Reaction Mixture Preparation and Incubation

The biodegradation potential of partially purified rhodanese toward cyanide-based pesticides was evaluated by measuring the decline in free cyanide after incubation with the enzyme extract. Reaction mixtures (1.5 mL) contained pesticide solution of known cyanide content, rhodanese extract, 50 mM sodium thiosulfate, and 100 mM phosphate buffer (pH 7.5–8.5). Three controls were included: a no-enzyme control, an enzyme blank, and a heat-inactivated enzyme control (boiled 10 min). Tubes then, were incubated at 37 °C for 30–120 min, after which reactions were stopped with 1% formaldehyde, cooled on ice, centrifuged at 10,000× *g* for 5 min, and the supernatants collected for cyanide analysis [39].

#### 4.9.2. Determination of Remaining Cyanide (König Reaction)

Residual cyanide was quantified using a modified König (pyridine–pyrazolone) colorimetric assay. To 500 µL of supernatant, 50 µL chloramine-T was added and allowed to react for 2 min, followed by addition of the pyridine–pyrazolone reagent. Samples were incubated at room temperature for 5–10 min to develop the red chromophore, and absorbance was measured at 578 nm using a UV–Vis spectrophotometer (UV-1800, Shimadzu, Kyoto, Japan).Cyanide concentrations were calculated from a freshly prepared KCN standard curve [60].

#### 4.9.3. Calculation of Cyanide Degradation Efficiency

Biodegradation efficiency was expressed as the percentage reduction of cyanide relative to the negative control. Cyanide concentration in each sample (*C_t_*) was interpolated from the standard curve, and degradation efficiency (%) was computed using

$$Cyanide\;degradation\;\left(\%\right)=\left(\frac{C_{0}-C_{t}}{C_{0}}\right)\times 100$$

where $C_{0}$ is the cyanide concentration in the negative control and $C_{t}$ is the residual cyanide after enzyme treatment. All measurements were performed in duplicate, and mean values were reported.

### 4.10. Statistical Analysis of Experimental Data

All comparative and optimization experiments were conducted in triplicate (*n* = 3), and the results are reported as mean values with their corresponding standard deviations. Statistical analysis was performed using GraphPad Prism (version 9.5.1; GraphPad Software, Boston, MA, USA). Differences between experimental conditions were evaluated by one-way analysis of variance, with *p* values below 0.05 considered statistically significant.

## 5. Conclusions

Rhodanese produced by *Kocuria rhizophila* exhibits strong catalytic efficiency, broad pH and temperature tolerance, and effective detoxification of multiple cyanide forms, highlighting its potential as a biocatalyst for environmental remediation. Its production behavior and biochemical properties align well with those of established microbial rhodaneses, supporting the reliability of this less-explored source. Nonetheless, because detoxification was evaluated under controlled laboratory conditions, the enzyme’s performance in complex cyanide-contaminated matrices remains uncertain, warranting future investigations under realistic environmental conditions and at applied process scales.

## Figures and Tables

**Table 1:** Overall purification profile of Crude Rhodanese Obtained from *Kocuria rhizophila*.

Purification Steps	Total Protein (mg/mL)	Total Activity (µmol/min)	Specific Activity (µmol/mg/min)	Yield %	Purification Fold
Crude	1.275	132.30	2.17	100	1.00
Acetone	1.052	62.41	3.92	48.29	1.81
Ammonium sulfate	1.010	58.67	2.82	9.63	1.30
CM sephadex C 50 ion exchange chromatography	0.270	19.56	9.83	2.73	4.53
Sephadex G-100 gel filteration	0.050	4.54	11.56	12.78	5.33

Values represent results from a representative purification procedure.

**Table 2:** Kinetic parameters (Km and Vmax) of rhodanese determined using KCN and Na_2_S_2_O_3_ as substrates.

Substrate	Km (mM)	Vmax (RU/mL/min)
KCN	33.92 ± 0.11	5.60 ± 0.03
Na_2_S_2_O_3_	19.65 ± 0.24	5.84 ± 0.014

Values are expressed as mean ± SD of independent experiments.

**Table 3:** Effect of selected metal ions on rhodanese activity.

Metals	Rhodanese Activity (%) Under Different Concentration (1.0, 5.0, 10.0 mM)		
	1.0 mM	5.0 mM	10.0 mM
Control	100.00	100.00	100.00
KCl	70.75 ± 0.35	64.19 ± 0.87	61.05 ± 0.32
MgCl2	92.15 ± 0.21	82.16 ± 0.20	75.00 ± 0.65
BaCl2	80.20 ± 0.28	87.32 ± 0.94	91.73 ± 0.80
NiCl2	83.45 ± 0.35	91.00 ± 0.38	95.00 ± 1.02
MnCl2	78.93 ± 0.39	88.64 ± 0.76	93.81 ± 0.98
SnCl2	87.75 ± 0.35	93.00 ± 1.02	98.58 ± 0.10
NaCl	88.46 ± 0.20	45.76 ± 0.24	18.50 ± 0.31

Values are expressed as mean ± SD (n = 3).

**Table 4:** Substrate specificity of rhodanese toward different sulfur compounds.

Sulphur Compounds	Specificity (%)
Sodium thiosulphate (Na_2_S_2_O_3_)	100.0 ± 0.00
Sodium metabisulphite (Na_2_S_2_O_5_)	15.4 ± 0.28
Ammonium persulphate ((NH_4_)_2_S_2_O_8_)	24.15 ± 0.21
2-mercaptoethanol (CH_2_(SH)CH_2_(OH))	18.0 ± 0.00
Sodium sulfite	19.80 ± 0.14

Values are expressed as mean ± SD of independent experiments.

## Data Availability

The data supporting the findings of this study are available within the article and its Supplementary Materials. Additional information may be obtained from the corresponding author upon reasonable request.

## Supplementary Materials

The following supporting information can be downloaded at: https://www.mdpi.com/article/10.3390/molecules31060915/s1, Figure S1: Morphological identification of the *Kocuria rhizophila* isolate; Figure S2: VITEK biochemical identification profile of *Kocuria rhizophila*; Figure S3: Agarose gel electrophoresis of amplified 16S rRNA gene fragments of *Kocuria rhizophila*; Figure S4: 16S rRNA multiple sequence alignment demonstrating high nucleotide identity between the isolate and reference *Kocuria rhizophila* sequences; Figure S5: Pairwise comparison of 16S rRNA gene sequences showing percent identity and divergence between the isolate and reference *Kocuria* species.
