# Supplementary material for: Biodegradation of Cyanide-Based Compounds by Rhodanese Produced from Kocuria rhizophila Under Submerged Fermentation and Its Role in Environmental Detoxification
Source: Molecules. 2026 Mar 10;31(6):915. doi: 10.3390/molecules31060915 (PMC13029668; doi:10.3390/molecules31060915)
Supplement: Supplementary file 1 [file molecules-31-00915-s001.zip › molecules-4127599-supplementary.pdf]

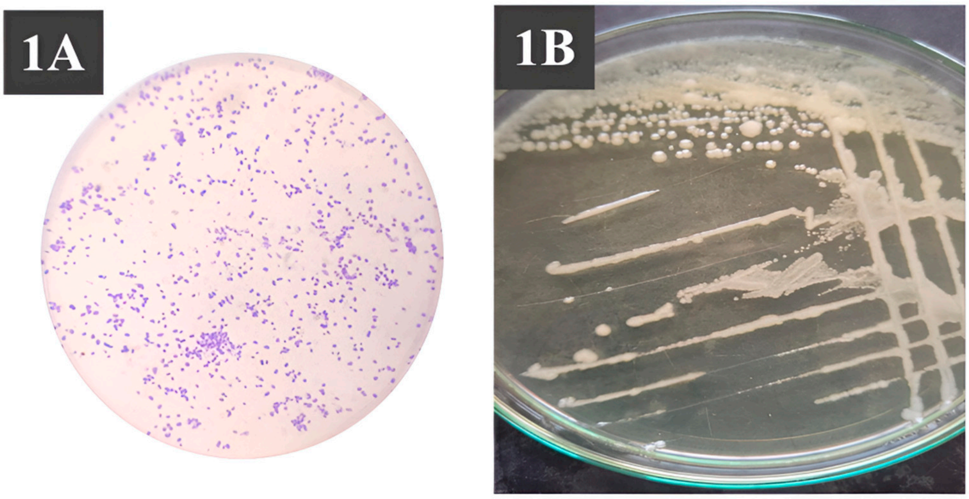

Figure S1. Morphological identification of the *Kocuria rhizophila* isolate. **(A)** Microscopic appearance of the isolate observed under light microscopy, showing coccoid cell morphology and characteristic cellular arrangements. **(B)** Macroscopic colony morphology of the isolate grown on agar medium, illustrating colony shape, surface features, and pigmentation.

bioMérieux Customer: Microbiology Chart Report Printed December 21, 2022 8:08:21 AM AST

Patient Name: 20, . Patient ID: tjngfhd
Location: Physician:
Lab ID: 49 Isolate Number: 1

Organism Quantity:
Selected Organism : Kocuria rhizophila

Source: **Collected:**

**Comments:**	

**Identification Information**	**Analysis Time:**	7.80 hours	**Status:**	Final
**Selected Organism**	98% Probability<br>**Bionumber:**	**Kocuria rhizophila**<br>010010302000000		
**ID Analysis Messages**				

Biochemical Details

2	AMY	-	4	PIPLC	-	5	dXYL	-	8	ADH1	+	9	BGAL	-	11	AGLU	-
13	APPA	-	14	CDEX	-	15	AspA	-	16	BGAR	-	17	AMAN	-	19	PHOS	-
20	LeuA	+	23	ProA	-	24	BGURr	-	25	AGAL	-	26	PyrA	-	27	BGUR	-
28	AlaA	+	29	TyrA	+	30	dSOR	-	31	URE	-	32	POLYB	-	37	dGAL	-
38	dRIB	-	39	lLATk	+	42	LAC	-	44	NAG	-	45	dMAL	-	46	BACI	-
47	NOVO	-	50	NC6.5	-	52	dMAN	-	53	dMNE	-	54	MBdG	-	56	PUL	-
57	dRAF	-	58	O129R	-	59	SAL	-	60	SAC	-	62	dTRE	-	63	ADH2s	-
64	OPTO	-															

Figure S2. VITEK biochemical identification profile of *Kocuria rhizophila*.

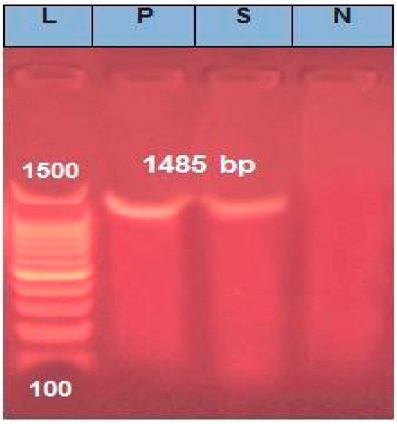

Figure S3. Agarose gel electrophoresis of amplified 16S rRNA gene fragments of Kocuria rhizophila. Lane L: 100 bp DNA ladder. Lanes P and S show a single amplicon of approximately 1485 bp, corresponding to the expected size of the bacterial 16S rRNA gene.

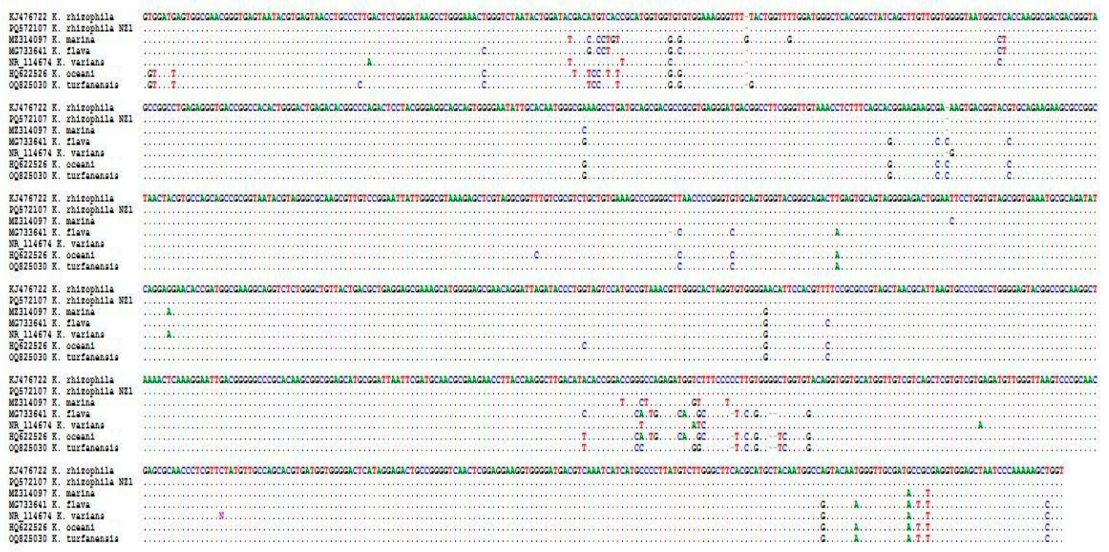

Figure S4. 16S rRNA multiple sequence alignment demonstrating high nucleotide identity between the isolate and reference *Kocuria rhizophila* sequences.

Percent Identity

Divergence

	1	2	3	4	5	6	7	8	9	10	11	12	13	14	15	16	17	18	19	20	21	22	23	24		
1		99.8	99.4	99.7	99.7	99.0	99.7	99.7	99.7	97.4	97.8	97.8	96.9	96.2	96.4	98.6	96.2	96.1	96.8	98.3	95.9	95.5	97.7	96.4	1	EU660422 K. rhizophila ID4123
2	0.0		99.7	99.9	99.9	99.2	99.9	99.9	99.9	97.5	97.9	97.9	97.1	96.4	96.6	98.8	96.4	96.2	97.0	98.4	96.1	95.7	97.8	96.6	2	OM585539 K. rhizophila LSH32
3	0.1	0.1		99.6	99.6	98.8	99.6	99.6	99.6	97.2	97.6	97.6	96.9	96.0	96.4	98.7	96.1	95.9	96.7	98.3	95.9	95.5	97.7	96.3	3	OM585512 K. rhizophila LAA5
4	0.1	0.1	0.2		100.0	99.1	100.0	100.0	100.0	97.6	98.0	97.9	97.2	96.4	96.5	98.8	96.4	96.2	96.9	98.5	96.0	95.7	97.9	96.5	4	NR_026452 K. rhizophila TA68
5	0.1	0.1	0.2	0.0		99.1	100.0	100.0	100.0	97.6	98.0	97.9	97.2	96.4	96.5	98.8	96.4	96.2	96.9	98.5	96.0	95.7	97.9	96.5	5	KJ476722 K. rhizophila DSM 11926
6	0.8	0.8	0.9	0.9	0.9		99.1	99.1	99.1	97.3	97.5	97.9	96.4	96.7	96.5	98.3	96.4	96.0	97.3	98.0	96.1	95.7	97.5	96.6	6	AP009152 K. rhizophila DC2201
7	0.1	0.1	0.2	0.0	0.0	0.9		100.0	100.0	97.6	98.0	97.9	97.2	96.4	96.5	98.8	96.4	96.2	96.9	98.5	96.0	95.7	97.9	96.5	7	KT387335 K. rhizophila NT_92
8	0.1	0.1	0.2	0.0	0.0	0.9	0.0		100.0	97.6	98.0	97.9	97.2	96.4	96.5	98.8	96.4	96.2	96.9	98.5	96.0	95.7	97.9	96.5	8	KP345929 K. rhizophila 3330
9	0.1	0.1	0.2	0.0	0.0	0.9	0.0	0.0		97.6	98.0	97.9	97.2	96.4	96.5	98.8	96.4	96.2	96.9	98.5	96.0	95.7	97.9	96.5	9	PQ572107 K. rhizophila NZ1
10	2.2	2.3	2.4	2.2	2.2	2.6	2.2	2.2	2.2		99.4	97.7	96.8	96.6	96.4	97.3	96.4	96.1	96.9	97.3	96.1	95.9	99.1	96.5	10	NR_027193 K. carniphila
11	1.8	1.9	2.0	1.8	1.8	2.3	1.8	1.8	1.8	0.6		97.8	97.2	96.8	96.5	97.7	96.6	96.4	97.0	97.7	96.2	96.0	99.2	96.8	11	KR085939 K. gwangalliensis
12	1.9	2.0	2.1	2.0	2.0	2.0	2.0	2.0	2.0	2.0	2.0		96.0	96.1	96.4	97.8	96.4	96.6	96.8	97.8	95.6	95.9	97.3	96.2	12	MZ314097 K. marina
13	2.7	2.7	2.6	2.6	2.6	3.4	2.6	2.6	2.6	3.1	2.7	3.7		96.5	96.8	96.9	96.5	96.3	96.9	97.0	96.8	96.2	97.3	96.9	13	FR691399 K. palustris
14	2.8	2.9	3.0	2.9	2.9	2.6	2.9	2.9	2.9	2.7	2.6	3.3	2.7		95.9	95.9	96.4	95.8	97.0	96.0	95.8	95.4	96.4	96.5	14	NR_144586 K. subflava
15	3.1	3.1	3.0	3.2	3.2	3.2	3.2	3.2	3.2	3.4	3.3	3.3	3.1	3.3		96.7	97.8	96.3	97.8	96.4	98.6	95.9	96.8	98.0	15	MG733641 K. flava
16	1.1	1.1	1.0	1.2	1.2	1.6	1.2	1.2	1.2	2.5	2.1	2.0	2.8	3.3	2.9		96.0	96.2	96.9	98.9	95.9	95.9	97.8	96.2	16	MW301608 K. salsicia
17	3.4	3.4	3.5	3.4	3.4	3.4	3.4	3.4	3.4	3.5	3.3	3.3	3.3	2.9	2.1	3.7		96.4	98.8	96.1	97.7	95.7	96.4	98.5	17	MF769334 K. dechangensis
18	3.4	3.5	3.6	3.5	3.5	3.7	3.5	3.5	3.5	3.7	3.4	3.2	3.5	3.6	3.5	3.5	3.5		96.9	95.9	95.9	97.3	96.4	96.2	18	NR_169461 K. massiliensis
19	2.8	2.8	2.9	2.9	2.9	2.6	2.9	2.9	2.9	3.0	2.9	3.0	3.0	2.3	2.1	2.8	1.2	3.1		96.8	97.8	96.0	96.9	98.3	19	KM186611 K. polaris
20	1.5	1.5	1.5	1.4	1.4	2.0	1.4	1.4	1.4	2.5	2.1	2.0	2.7	3.2	3.2	0.9	3.6	3.8	3.0		95.8	95.8	97.6	96.2	20	NR_114674 K. varians
21	3.6	3.7	3.6	3.8	3.8	3.7	3.8	3.8	3.8	3.8	3.7	4.2	3.2	3.5	1.4	3.9	2.2	4.1	2.2	4.0		95.4	96.8	98.9	21	HQ622526 K. oceani
22	3.9	3.9	3.8	3.9	3.9	3.9	3.9	3.9	3.9	3.8	3.6	3.7	3.4	3.9	3.7	3.6	4.1	2.7	3.8	3.7	4.4		96.4	95.6	22	MG948160 K. kristinae
23	1.9	2.0	1.9	1.9	1.9	2.3	1.9	1.9	1.9	0.9	0.8	2.5	2.6	3.0	3.0	1.9	3.5	3.4	3.0	2.1	3.1	3.3		96.8	23	NR_116744 K. atrinae
24	3.1	3.2	3.3	3.3	3.3	3.2	3.3	3.3	3.3	3.4	3.1	3.5	3.0	2.7	2.0	3.5	1.4	3.7	1.6	3.5	1.1	4.2	3.1		24	OQ825030 K. turfanensis
	1	2	3	4	5	6	7	8	9	10	11	12	13	14	15	16	17	18	19	20	21	22	23	24		

Figure S5. Pairwise comparison of 16S rRNA gene sequences showing percent identity (upper triangle) and divergence (lower triangle) between the isolate and reference *Kocuria* species.
